# Ethical aspects of brain computer interfaces: a scoping review

**DOI:** 10.1186/s12910-017-0220-y

**Published:** 2017-11-09

**Authors:** Sasha Burwell, Matthew Sample, Eric Racine

**Affiliations:** 1 Neuroethics Research Unit, Institut de recherches cliniques de Montréal, 110 avenue des Pins Ouest, H2W lR7, Montréal, QC Canada; 2 Department of Experimental Medicine, McGill University, Montréal, Canada; 3 Department of Medicine and Department of Social and Preventative Medicine, Université de Montréal, 110 avenue des Pins Ouest, H2W lR7, Montréal, QC Canada; 4 Departments of Neurology and Neurosurgery, Experimental Medicine and Biomedical Ethics Unit, McGill University, 110 avenue des Pins Ouest, H2W lR7, Montréal, QC Canada

**Keywords:** Brain-computer interface, Brain-machine interface, Scoping review, Ethics of technology

## Abstract

**Background:**

Brain-Computer Interface (BCI) is a set of technologies that are of increasing interest to researchers. BCI has been proposed as assistive technology for individuals who are non-communicative or paralyzed, such as those with amyotrophic lateral sclerosis or spinal cord injury. The technology has also been suggested for enhancement and entertainment uses, and there are companies currently marketing BCI devices for those purposes (e.g., gaming) as well as health-related purposes (e.g., communication). The unprecedented direct connection created by BCI between human brains and computer hardware raises various ethical, social, and legal challenges that merit further examination and discussion.

**Methods:**

To identify and characterize the key issues associated with BCI use, we performed a scoping review of biomedical ethics literature, analyzing the ethics concerns cited across multiple disciplines, including philosophy and medicine.

**Results:**

Based on this investigation, we report that BCI research and its potential translation to therapeutic intervention generate significant ethical, legal, and social concerns, notably with regards to personhood, stigma, autonomy, privacy, research ethics, safety, responsibility, and justice. Our review of the literature determined, furthermore, that while these issues have been enumerated extensively, few concrete recommendations have been expressed.

**Conclusions:**

We conclude that future research should focus on remedying a lack of practical solutions to the ethical challenges of BCI, alongside the collection of empirical data on the perspectives of the public, BCI users, and BCI researchers.

**Electronic supplementary material:**

The online version of this article (10.1186/s12910-017-0220-y) contains supplementary material, which is available to authorized users.

## Background

Brain-Computer Interface (BCI) is a rapidly developing area of neuroscience research. As such, there is no consensus on a definition of BCIs. For example, some researchers include stimulating devices, such as cochlear implants, in their definition, while others do not [1–9]. However, there are a few elements upon which researchers and scholars commonly agree. These crucial elements are the ability of a BCI to (1) detect brain activity directly, (2) provide feedback in real-time or near-time, (3) classify brain activity, and (4) provide feedback to the user that reflects whether she/he successfully attained a goal [10]. Similar to the majority of authors [9], we consider BCI to be a device that detects brain signals conveying intention and translates them into executable output by a machine [11]. In other words, it is a “direct connection between living neuronal tissue and artificial devices that establishes a non-muscular communication pathway between a computer and a brain” [12]. BCIs have the potential for great significance in the daily lives of patients [13]. For example, BCIs can be used as “spellers” for individuals who have no other way of communicating [12, 14–18], can give people who are locked-in or paralyzed some control over their environment [12, 15, 19–24], and may be able to aid rehabilitation after spinal cord injury via artificial stimulation of muscles [11, 24], among other potential applications.

There are three main approaches to recording brain signals for use with BCI. (1) *Noninvasive* recording methods record signals from the scalp; these include electroencephalogram (EEG), functional magnetic resonance imaging (fMRI), and near-infrared spectroscopy (NIRS). *Invasive* recording is done either (2) by electrocorticography (ECoG), where signals are recorded from the surface of the cortex or (3) from within the cortex itself with the help of microelectrode arrays. The signal-to-noise ratio improves as the methodology becomes more invasive; however, invasive BCIs have more associated risks than their noninvasive counterparts due to, among other things, the need for surgery and its attendant risks or possible glial scarring [25]*.* Therefore, EEG, despite its lower signal-to-noise ratio, is quite popular for use with BCI given its safety, portability, cost-effectiveness, and high temporal resolution [14]. At the moment, only electrical signals are likely to be of practical value. However, other signal types (such as fMRI) could provide improved spatial resolution [26].

In addition to the various recording paradigms, there are a few different possibilities for signal generation, i.e., how much “will” needs to be exerted by the user to produce a signal for the control system to read [10]. *Spontaneous* or *active* BCI systems require the user to generate certain brain patterns. *Evoked* BCI systems present external stimuli to the user, who is required to willfully attend to one of the stimuli. The last BCI system uses *passive* signal generation, in which ongoing brain activity such as arousal level is recorded [10].

The main proposed and most widely researched BCI use is as an assistive technology. Multiple studies have shown that BCI technology could give locked-in patients the power to communicate again with a BCI speller [15]. Similarly, those who are paralyzed could use their brain signals to control prosthetic limbs [15, 27], cursors [15], and wheelchairs [12]. Other BCI uses include entertainment, such as video games [23], and enhancement, including potential military surveillance applications [22]. Despite intense research, BCI development is not at a point where neural devices can be reliably used as therapy, entertainment, or enhancement. With current technology, if an individual retains any muscle control, muscular-based communication and motor systems are more effective and efficient than BCI [5]. In addition, complete locked-in patients – those who have no remaining muscle movement – cannot use BCI for unknown reasons [5]. Despite these limitations, BCI is regularly researched, tested on patients, and is even being marketed to the public – for example, wireless EEG headsets for personal monitoring of cognitive health (https://www.emotiv.com/), and EEG-based spelling and painting systems (http://www.intendix.com/). Some forms of BCI are likely to be expensive, posing questions of affordability and coverage under health care plans [28]. In addition, the regulatory issues involved with medical devices make it more financially feasible for companies to focus on consumer devices instead [28], which raises the question of whether this will limit the ability of people with severe disabilities to access BCI as assistive technology. This is only one of many challenges likely to accompany BCI research and development.

The transformative technological potential cited by BCI researchers would seem to necessitate equally significant ethical inquiries. But similar to the disagreement over the definition of BCI, there is also controversy with respect to its ethical implications. Some argue that ethical issues associated with BCIs are no different than those associated with other medical technologies [2, 10], while others advance that the “use of BCI is the greatest ethical challenge that neuroscience faces today” [4]. We do not intend to address that debate directly in this article. Regardless of whether the challenges presented are unprecedented or not, there clearly are some ethical considerations related to the use of BCIs that will need to be addressed by researchers and research participants, and eventually by clinicians, patients, and society at large if the technology moves forward. To help chart this literature, we undertook a scoping review to provide an overview of ethical issues associated with BCIs. The identification of key topics discussed as well as qualitative characterization of their content should be useful to bioethics researchers who wish to build from this review to create research tools (e.g., questionnaires, interview grids, surveys), as well as to researchers in science, engineering, or medicine who would like to better understand the current literature.

## Methods

We performed a scoping literature review, specifically Levac et al.’s [29] update of the method proposed by Arksey and O’Malley [30]. We chose to use this method of review because of its applications for summarizing findings, exploring the extent of research on a certain topic, and identifying research gaps. This review framework includes six stages: (1) identifying the research question, (2) identifying relevant studies, (3) study selection, (4) charting the data, (5) collating, summarizing, and reporting the results, and (6) consultation.

1. *Identifying the research question*

Our research goal was to analyze the literature on the ethics of BCIs in terms of the dominant issues discussed. By highlighting the coverage or lack of coverage in the literature, answering this question could inform recommendations for future research, as well as on-going development of neural technologies.

2. *Identifying relevant studies and 3. Selecting studies for inclusion*

We conducted two consecutive PubMed searches, one general and one issue-oriented. PubMed was chosen because of its wide range of literature specifically on biomedical devices like BCIs, and on their application for clinical or experimental purposes. The primary search occurred originally on June 6, 2016, was repeated on August 12, 2016, and used the keywords related to the domain of ethics in general ((“brain computer interface” OR “BCI” OR “brain machine interface” OR “Brain-computer Interfaces”[Mesh]) AND (“ethics” OR “Ethics”[Mesh])) (*N* = 29). Articles were included if they (1) were written in English, (2) presented conceptual discussions or empirical findings on ethics of BCI, (3) referred to humans, and (4) consider BCI as technology that records directly from the brain to create executable output. We excluded articles that were (1) in a language other than English, (2) related solely to deep brain stimulation, or other brain stimulation technology, or (3) primarily focused on technical or engineering aspects of BCI. After applying these criteria, 24 articles remained from the primary search.

**Fig. 1:**
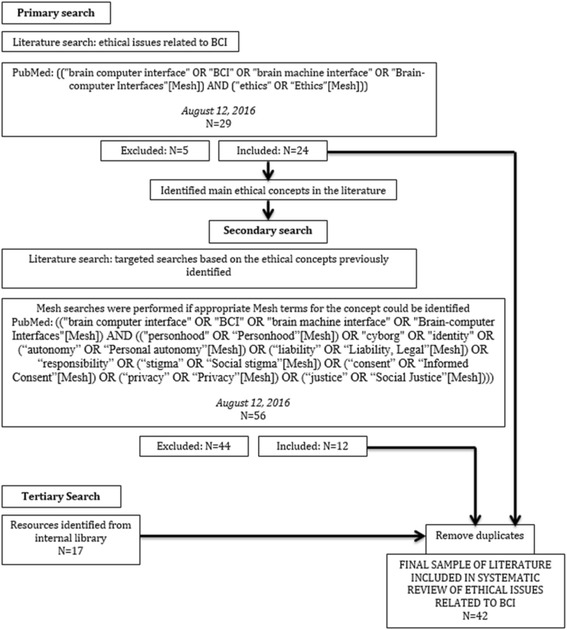
Search Strategy

From the primary search, we identified a list of issues frequently discussed in the ethics literature on BCI (Fig. 1); from these topics, we generated keywords and performed a secondary targeted search to include articles that are framed in terms of a specific topic within the domain of ethics. Mesh terms were used when they were relevant to the topics of interest. This secondary search occurred on August 12, 2016, with the keyword ((“brain computer interface” OR “BCI” OR “brain machine interface” OR “Brain-computer Interfaces”[Mesh]) AND ((“personhood” OR “Personhood”[Mesh]) OR “cyborg” OR “identity” OR (“autonomy” OR “Personal autonomy”[Mesh]) OR (“liability” OR “Liability, Legal”[Mesh]) OR “responsibility” OR (“stigma” OR “Social stigma”[Mesh]) OR (“consent” OR “Informed Consent”[Mesh]) OR (“privacy” OR “Privacy”[Mesh]) OR (“justice” OR “Social Justice”[Mesh]))) (*N* = 56). After applying the same inclusion and exclusion criteria as the primary search to the 56 articles the secondary search yielded, 12 articles remained. And, after excluding duplicate articles from the primary and secondary searches, we were left with a total of 27 articles.

Following the primary and secondary searches, we found further relevant sources (*N* = 17) by consulting our internal library and relevant articles referenced within the initial sample. After excluding duplicates shared with the initial searches, we had a final sample of *N* = 42 articles for analysis.

4. *Charting the data*

We chose to extract information relating to the type of article; an article is categorized as “empirical” if it relies primarily on collected data on social dynamics or attitudes (e.g., surveys, interviews, etc.). Other, non-empirical articles include commentaries, reviews, and discussion articles. In addition, we noted the specific ethical issues covered in each article, whether mentioned briefly or discussed in-depth. MS conducted a content analysis using NVivo9, while SB extracted the relevant content from each article using an Excel table organized by conceptual issue in collaboration with ER. MS and SB then compared their lists of issues, discussed differences, and reached a consensus. The focus was on frequently discussed issues, though multiple rarer issues were observed, as we discuss in a later section. This final categorization was then used to create a table (see Additional file 1) that characterizes each source according to the issues mentioned, whether in passing or in depth.

5. Collating/summarizing/reporting results

As Arksey and O’Malley originally suggested, we present our findings in a narrative fashion, supported by descriptive numerical summaries containing key characteristics about the articles included [30]. Once the content had been extracted from the articles, SB and MS determined the main conclusions within each ethical concept and the justifications presented for each. ER reviewed extracted content and provided feedback on its organization.

6. Consultation

External review of our findings was provided through consultation with four experts in the topic area, representing clinical medicine, biomedical engineering, bioethics, and end-user perspectives. Consultants were asked to read a late-stage draft of study results and to comment on accuracy, clarity, and comprehensiveness of results. Their feedback was used to revise the manuscript along these dimensions, most significantly in more precisely presenting study methods and limitations, in refining our explanations of the content of each ethical issue, and in noting several potentially significant ethical issues that were not found during content analysis.

## Results

### Sample characteristics

The review of collected sources revealed that ethical issues are broadly distributed across the literature, and that most articles detail more than one issue and mention many more in passing. This finding is partially explained by the many overview pieces that are published in the topic area. Empirical articles, moreover, represented a minority of coded articles (*N* = 11). The most frequently cited issues include user safety (*N* = 24), justice (*N* = 20), privacy and security (*N* = 19), and balance of risks and benefits (N = 19).^1^

### User safety and risk-benefit analyses

Among all the concerns surveyed in the literature, the most commonly mentioned issues encompass the safety of BCI devices and the related balance of risk and benefit to the BCI user. These dimensions of concern are consistent with what one would expect from any new biomedical device. In terms of safety, authors assert that BCIs may pose direct risk of harm to the user, especially for devices that require surgical interventions. For devices that must be implanted under the skin or skull, potential complications include infection of the surrounding tissue and acute trauma to the brain [26], among others. For long-term implants, the affected neural tissue may also develop glial scarring, which can surround the implant and impede BCI function [25, 31]. Even non-invasive devices may pose serious risks of harm; some authors wonder whether the brain’s plasticity in still-developing children and even in adults could bring about unknown negative side-effects of BCI use [17]. And the unknown reversibility of these side-effects presents yet another worry: would the brain or the user return to normal after a BCI is removed? These concerns, though frequent, are often only acknowledged and not analyzed further.

Non-medical safety issues are also discussed. Some authors stress the potentially serious harms of intense training and cognitive concentration for would-be BCI users. The need for regular and challenging training sessions may impose physical, emotional, and financial burdens on the user and their family [21]. And a BCI used to control a motor prosthetic, for example, may require more cognitive planning and attention than a user can achieve on a regular basis, leading to frustration [32]. Device failure, similarly, may place the user in particularly difficult situations – for example, a BCI wheelchair failing as its user is crossing a street could have deadly consequences. Just as a BCI can provide perhaps unprecedented benefit to persons with locked-in syndrome, a sudden lack of functionality would impact the user directly and immediately [6]. As users become increasingly dependent on the technology, partial device failures or errors become more significant.

These judgments or questions of BCI safety were often featured in higher-level discussions of the relative risks and benefits of BCI devices. Many authors either addressed this balance explicitly or stressed the need for risk-benefit analysis. Such evaluation, for example, allows for the comparison of BCIs with alternative assistive technology [33] or the determination of their appropriateness for a given patient [26]. These crucial analyses may not yet be possible, however, given several epistemic obstacles. General scientific uncertainty plays a role [5], but several authors note a lack of data or studies on the relative benefits of BCI [6, 16, 25]. Therefore, the very idea of an acceptable expectation of benefit may currently be unrealistic [5]. Nevertheless, many authors do not hesitate to describe BCI as an inherently risky technology, given the range of foreseeable and unforeseeable harms described above.

### Humanity and personhood

BCI involves a direct interaction between brains and machines, and this interaction brings with it a series of questions regarding its effect on humanity and personhood along several dimensions. In a more philosophical mode, authors debate whether BCIs become part of the user’s “body schema.” The question – is it a tool or is it myself? – takes on an ethical valence when researchers ask whether BCI users will become “cyborgs.” The Oxford English Dictionary defines a cyborg as “…a person whose […] capabilities are extended beyond normal human limitations by a machine; an integrated man-machine system”. Not all authors are convinced that this concern is unique to BCIs, that it is worrying, or that it is even possible. Some emphasize the fact that we already have used technology to tinker with ourselves – specifying examples such as sports equipment and other medical interventions – and thus humans are already intricately linked to their technologies [9]. We incorporate tools into our self-understanding and body schemas [6, 8], and routinely use technology to change the body in the form of artificial devices replacing broken parts [9].

In contrast, others are quite concerned about the potential of BCI to impact our “humanity.” Demetriades et al. argue that being more robotic makes one less human, that BCI could generate the “risk of losing what makes us human” [4]. This is sometimes explained in terms of the unprecedented direct contact between brain and machine that is inherent to BCI [6]. On another note, Zehr believes that we could actually overcome the limitations of our species, evolve into a “*Homo sapiens technologicus”* that uses technology to enhance its functioning [34]. In addition to this, research has found that BCI users are not entirely comfortable with the idea of ‘cyborgization’: interviewed BCI users tended to distance themselves from the idea of becoming a functional man-machine hybrid [35].

Aside from ‘cyborgization’ and issues of affected humanity, concerns related to personhood are also debated. As indicated by the phenomena of interest, including changes in social identity, personality, and authenticity, the understanding of personhood in this literature is not narrowly Kantian. The focal point is thus not an individual’s capacity to reason, but rather reflects a broader relational perspective on the constitution of the person. Beyond that commonality, there remains disagreement. Some argue that identity changes are not worth discussing from an ethical standpoint. Patients themselves tend to not worry about changes in identity, as the chronic illness they face has already created many radical identity changes [9]. Other authors note that our identity fluctuates naturally, and can be changed by other medical therapies such as medication or even by having a glass of wine or going on vacation [36]. On the other side of the debate, authors assert that BCI may change our social identity, body schema [8], or individual psychological aspects [10]; they argue that the potential for BCI to induce widespread plastic changes in the brain [26] is something that needs consideration. There is also some public concern that people with brain implants would have a change in character, that they would no longer be “themselves” [7]. Outside of this argument is the belief that questions of identity should not be brought into the ethical debate at all [3]: as one researcher suggested, “It would be beyond arrogant to tell [BCI users] ‘I think this might change your identity, so I am not allowing you to use this technology’” [10]. Hence, in the literature, the debate is not so much over whether BCI will cause identity changes, but over whether those changes in personal identity are a problem that should impact technological development or access to BCI.

Lastly, the capability of BCI to allow communication in locked-in patients creates hope for restoration of personhood. Certain criteria of personhood include communication (for example, Fletcher’s 15 ‘indications’ for personhood [37])^2^ and it is suggested that loss of speech, due to resulting social isolation could lead to potential loss of personhood in that individual [38]. Because of this, a BCI that enables communication also enables greater social inclusion [39], and could save or restore personhood in someone who is losing the ability to interact with their loved ones and community. Even non-assistive technology BCI, such as that used for entertainment, could improve social access and expressive potential in the user [13].

### Stigma and normality

Another ethical theme encountered regularly in the literature is whether BCI has the potential to influence or be influenced by the social stigma of disability. There is concern within the literature that individuals could be influenced to seek out BCI because of disease stigma [25] or the negative idea that persons with disability are a burden on society [9]. This becomes even more of an issue when quality of life is brought into play. Most BCI researchers [10] and clinicians [21] assume that BCIs, as an assistive technology, will increase quality of life for people with disabilities and their loved ones. While this may be true from a narrowly medical perspective, a BCI device might ultimately increase the stigma of disability associated with an individual, which could influence potential users to not use BCI in spite of its potential benefits [25, 39, 40].

Restoration of “normal” abilities is one of the main uses of BCI cited in the literature [41]. Jebari and Hansson (2013) interviewed some members of the public who felt that BCI would make individuals with disability more ‘normal’ and able to engage in social interactions [7]. While this could be argued for, it may be partly misguided, as some patients in particular do not see themselves as fitting into a “deficit model” of disability [9]. If an individual does not see him or herself as disabled, is a BCI that is meant to be assistive technology actually an enhancement? This raises questions as to what the end result of BCI should be. The definition of “treatment” tends to be benchmarked to the species-typical body [42], and the principle of beneficence suggests that doctors have an obligation to restore health to ‘normal’ levels [22]. However, there is also the perspective of “tyranny of the normal” [43], as described elsewhere by Anita Silvers.^3^ For example, some individuals in the deaf community view cochlear implants as an enhancement instead of a treatment [2]. In this case, becoming “‘[n]ormal’ may not be what all end-users want” [8]. Setting the standard for ‘normal’, and thus defining the line between treatment and enhancement, is a serious challenge and one which implicates adjacent ethical concerns, like the individual’s ability to autonomously determine their particular type of body or form of life.

### Autonomy

The concept of autonomy is overarching, and thus has implications for other key ethical themes including responsibility, informed consent, and privacy. However, it is also a central issue in and of itself, and is used across clinical and ethical discussions. We note that the term is used differently by ethicists than by engineers and neuroscientists.^4^ For ethicists, autonomy refers to an individual’s capacity to self-determine. In the context of BCIs, Glannon states that “nothing about the influence of neuromodulation on the brain and mind suggests that we should revise the concept of autonomy” in ethics; however, he also questions whether an action that is produced mostly or solely by a device can truly be attributed to a human [32]. He notes that, for example, if a BCI device has a causal role in decision making of the individual, this could negatively affect autonomy. To the same effect, the device may work too well: perhaps our normal system of brain to muscles to action has some inherent censoring properties, whereas BCI takes signal input directly from the brain and could result in inappropriate actions that would normally be considered but not actually executed [26]. Similarly, Vlek et al. found that the illusion of agency, where BCI users inaccurately claim to be the agent of action, is possible [44]. Overall, many but not all authors are concerned about possible side effects of BCI use on autonomy.

Just as frequently, authors discuss the possibility of increased autonomy via empowerment from the assistive applications of BCI (though not with regards to entertainment or enhancement purposes). The disorders towards which BCI has been targeted – amyotrophic lateral sclerosis, spinal cord injury, stroke, etc. – have profound impacts on motor and communication abilities. Several authors concur that BCI as assistive technology will empower patients by allowing increased independence and leading to an improved quality of life [9]. The disorders themselves undermine the autonomy of the individual by inhibiting the ability to act on one’s own desire. Therefore, the actual threat to autonomy exists because of the patient’s condition, and BCI alleviates this by allowing the patient to express his or her thoughts and behaviors [32]. In this way, BCI is regarded as instrumental to human dignity via the development of human agency [33].

### Responsibility

The potential widespread use of BCI raises interesting questions about moral and legal responsibility, including about whether we have less control over our thoughts than over our bodies [45], or whether the choice to get a BCI device makes the user responsible for all of the device’s output [8]. In essence, the argument is over whether the unique characteristics of BCI will require changes to our legal systems and understanding of morality.

On the one hand, the argument has been made that while BCI may be sophisticated, it is really no different from other technologies [2] and, as such, we need only fairly small legal adjustments to adequately address the liability issues associated with BCI [17]. Within this view, there are two suggestions. The first is that the BCI user should be held responsible for any unintended actions: these ethicists equate BCI use with the responsibility we ascribe to use of other potentially dangerous tools, such as cars [3], or the responsibility a parent has for the actions of their child and a dog-owner has for the actions of their dog [17, 33]. Indeed, in a survey of BCI researchers, Nijboer et al. found that the majority of respondents agree that BCI users are responsible for the executed actions and transmitted messages created with the aid of a BCI device [10]. The second suggestion is that unintended actions be considered a flaw of the device itself, and hence the economic burden of liability should be shifted onto BCI manufacturers, similar to how producers of goods are regularly held responsible for damaging events [17, 33].

On the other hand, there is also the view that current legal systems cannot appropriately deal with BCI use. O’Brolchain and Gordijn (2014) point out that, although in abstract BCIs seem no different than other tools, in reality they entail novel aspects that could affect attribution of responsibility to BCI users [45]. Demetriades et al. (2010) claim that “uncontrolled use of [BCI] threatens not only the ‘unwritten’ social norms, but also the ‘written’ laws in criminal justice” [4]. Others caution that we cannot conclude that observed machine behavior coincides with users’ endorsed actions [17], and that including computers in the decision-making process distorts our means of ascribing responsibility [45]. Specific aspects of BCI technology are cited as the cause of the difference in ascription: BCIs capture intent directly from the central nervous system, without the peripheral checks inherent in species-typical biological movement, and as a result actions might be triggered simply by subconscious events or passing thoughts [45]. In addition, the potential for a BCI device to be hacked, and thus have actions created by a third party, could impede the ascription of responsibility [25, 45]. In sum, with this view, current understandings of moral and legal responsibility are insufficient to deal with the use of BCI.

### Research ethics and informed consent

Another set of issues cluster around research ethics. Informed consent, as an issue, represents the dominant theme in terms of frequency and depth of coverage, but we also noticed several less common topics around the idea of researcher responsibility. Incidental findings, for instance, are mentioned as a potential challenge to researchers, who may find themselves with troubling or sensitive information about BCI users [25], whether about illnesses or psychological disposition. A properly functioning research team may also be a challenge; research teams must develop strategies for communication between interdisciplinary team members and with research participants in demanding environments [10], and also for distributing work and credit fairly [5]. Finally, multiple articles cited a researcher duty to foster public understanding of BCI technology in the face of inaccurate media coverage. Fulfilling this role may require researchers to, among other things, avoid “overhyping” the technology [5], develop relationships with the media [39], and train in new styles of communication [33]. Yet even when combined, these reflections on researcher responsibility receive only minor attention compared to the issue of informed consent.

We observe that BCI has triggered extensive ethical discussions of informed consent, perhaps because of perceived ethical difficulties that are specific to the technology and its target population. Farisco et al. (2015) note that informed consent must respect (1) disclosure (the patient has and understands all needed information), (2) capacity (ability of the patient to understand the information and make a reasonable decision), and (3) voluntariness (a decision made without coercion or influence) [46]. Many BCI end users are non-communicative patients, such as those in locked-in state, and thus have significantly impaired capacity to consent. Klein (2016) considers this a major issue of BCI use [25]; Haselager et al. suggest that assent to BCI is necessary if possible but is not sufficient for consent [5]. Overall, the majority of researchers agree that current benefits outweigh the risks of non-invasive BCI for LIS [10]. Despite this, currently accepted principles of informed consent suggest caution: some locked-in and non-communicative patients may not want BCI, despite its purported benefits. In addition, if a non-communicative individual has a BCI and can use it to achieve a basic level of communication, it is doubtful that this would be sufficient for informed consent for further research purposes [32]. These BCI users could still be highly vulnerable, and it would be difficult to ascertain if they retain the ability to make an informed decision, much less whether they can communicate that decision fully [46].

Similar concerns have been raised about which individuals are appropriate research subjects and about their capacity to consent. Patients with severe disability are susceptible to accepting increased risks, including surgical risks and cognitive impairment [2, 6, 25], in the hopes of some minimal benefits. As patients with quadriplegia, locked-in state, and other significant disabilities are the primary end users of BCI assistive technology, there are concerns that they could be choosing to use BCI and participate in BCI research out of desperation [5] or as a last resort [33]. Steps must be taken to ensure that voluntariness is not diminished by desperation, leading to inappropriate consent [5].

The voluntariness of patients’ consent could also be impacted by unrealistic expectations of benefit. At present, BCI is an experimental treatment and its therapeutic viability has not been proven [9, 22]. This could lead to therapeutic misconception in subjects [13, 25], where they expect to be cured by a technology that in reality has a 15–30% chance of not working at all for a given individual [25]; and subjects whose high expectations are not met could be at risk for depression [25]. This therapeutic misconception could be fed by the expectation gap created by the media [5, 25]. Journalistic channels, and even social media, are regularly used by scientists and researchers as a bridge to the public [47], but in this case Haselager et al. encourage more accurate communication with the media [5], and Tamburrini and Mattia caution that we must carefully ensure that the public does not develop unfounded expectations for current BCI technology [18]. At the moment, media coverage of BCI is extremely positive [25] and futuristic, often described as “mind reading” and a “cure” [5], which is a significant exaggeration of the capability of the technology. The over-expectations created by these two scenarios diminish the possibility of accurately understood disclosure by the subject, which could lead to consent that is not as informed as it should be.

### Privacy and security

With new ways to connect to the brain, there is a potential for new violations of user privacy. One study on public understandings of BCI revealed that privacy is a significant concern for participants [7]. Some scholars share that worry, suggesting that since BCI is capable of direct extraction of information from the brain, a subject may be “unaware of the extent of information that is being obtained from his or her brain” [13]. BCI devices could reveal a variety of information, ranging from truthfulness, to psychological traits and mental states, to attitudes toward other people [8], creating potential issues such as workplace discrimination based on neural signals [13, 17]. Currently, language and non-verbal communication act as chief mediators for understanding the content of another person’s mind, but as technology continues to develop, it is likely that we will see an increased capacity to observe others’ minds directly beyond the spectacular yet rudimentary feats currently accomplished [48].

A second privacy-related concern is hacking, i.e., an external source gaining control of a BCI device. Several authors noted that the use of wireless communication standards exposes BCI users to risk of interference from others [8, 25, 32]. Others speculate about the specific scenarios and identities of the malevolent actor, whether the government [45] or an unethical employer [7]. Beyond extracting information, harmful exploits could cause the BCI device to malfunction or allow it to be manipulated such that it harms the user [8, 49]. Bonaci et al. (2015) argue, based on these hypothetical scenarios, that BCI researchers should foster “neurosecurity,” analogous to similar efforts in computer science [49]. Overall, security and protection of privacy are deemed extremely important when considering implementation of BCI technology.

### Justice

A range of justice-related issues were identified in the literature, spanning the entire process of technological development from early design to distribution. Some scholars assert that, as BCIs are being engineered, those most likely to be affected by the technology, including potential end users [8, 50] and the general public [7], should have input into the design process. Wolbring et al. (2013) worry that most BCI literature treats disability as a medical issue rather than a socio-cultural one, suggesting that some perspectives of persons with disability have not been considered [42]. Many examples of BCI technology are currently still in the clinical research stage, so some justice concerns overlap with research ethics. Scholars ask what should happen to research subjects once a study is complete. Of particular concern is whether the participants keep the BCI device for their personal use at the end of the study and, if so, who is responsible for maintaining the technology [25]. As there can be risk of depression upon retraction of BCI from a user [33, 51], this is presented as a question that must be addressed.

Once BCIs become widely available, additional issues will arise. Looking ahead, scholars note the challenge of fair access, the need for which is stressed by patients and public alike [7, 9]. The potential for BCI devices to enhance a healthy user’s capabilities beyond “normal” may create social stratification [13] or unfairness between coworkers [8]. And for those who do opt to self-enhance, how long can they keep their advantage? Klein (2016) notes that, since invasive procedures are risky and can likely only be performed once in any given individual, we must reflect on the decision to give an individual an implantable device that will be possibly outdated and outperformed by other devices in the near future [25].

### Other issues

In our review, not all coded ethics concerns turned out to represent a general theme in the literature. These miscellaneous issues are either mentioned too infrequently or too briefly to be generalized. Military use, for example, is flagged occasionally as both a promising domain of application for neural technology [6, 22], as well as a morally questionable development [7, 52, 53]. The literature also contains occasional references to broader societal impacts, whether in terms of reactions towards religious or military uses of BCI [36], or in terms of changing social norms and “slippery slopes” [7]. Enhancement and transhumanism, too, are sometimes invoked by name [34, 35]. Authors also debated research priorities [13, 42]—should funds be directed towards the needs of individuals or populations, or towards neurological or social problems?—and referenced a need for guiding ethical principles [22, 31, 54] and new forms of regulation [1, 4, 49, 55].

Additional concerns were proposed to us by our expert consultants. Physical safety and user well-being, though not included in the original search strategy, was confirmed as a crucial topic. Consultants also noted the potentially misleading use of individual case studies in BCI research, alongside concerns regarding unpublished military research, unrealistic expectations from the user’s family, patient-caregiver or patient-family disagreements, and responsibility complications created by the use of “machine learning” [13]. In total, these “Other issues,” as listed here are not exhaustive but give a sense of the topics that are inconsistently discussed or resist generalization into the eight major themes described above.

## Discussion

The present review reveals general characteristics of the biomedical ethics literature and its major content foci, but before reflecting on those, several limitations of this study should be noted. Our study was conducted using a single database (i.e., Pubmed) most likely to include neural technology as an object of biomedical interest, searching primarily for terms (e.g., autonomy, privacy) that are frequently used in academic bioethics. A review based on other research databases and other non-ethics research domains would likely highlight different applications or definitions of BCIs, presenting the technology through alternative cultural or methodological lenses. Furthermore, the present review addresses only those issues that were frequently mentioned across the coded articles, with brief mention of other infrequently observed issues. Ethics concerns that are mentioned once or rarely, though underrepresented in our review, may be just as pressing as the eight categories above. As such, strong or weak representation in the results is not necessarily an indication of moral or regulatory significance. Despite these noted study limitations, there are several features of the literature sample that can be highlighted here.

In general, our results show that the ethics of BCI, broadly construed, receives significant attention in the academic biomedical ethics literature. Scholars have identified a wide range of concerns that, though perhaps not entirely unprecedented in bioethics, seem to warrant further attention in the context of neural technology and its development; BCI researchers in particular may find these preliminary findings useful in broadening the scope of engineering or design. However, the vast range of issues that are covered may also be a source of weakness for the literature. Many articles, perhaps to avoid missing a critical topic or to provide a general overview, attempt to address multiple ethical issues. As a result, the depth of discussion remains low throughout such articles. While overviews can justify a new research program or summarize an existing one, they may be less suitable for solving specific problems or for giving a thorough treatment to a single concern. This characteristic of the literature could partially explain our second observation.

Namely, there is a notable lack of recommendations in the literature for handling concrete ethical issues. While recommendations were not formally coded alongside ethical issues, we observed a predominance of discussion of issues over that of potential solutions or practical guidelines. BCI is clearly a quickly growing field with a potentially large consumer base, and while many have identified potential problems, few have made proposals to address these problems. Given that neural technology is currently being developed with or without direct acknowledgement of the above concerns, scholars deliberating on the ethics of BCI may need to better situate their work in systems of technology governance, either through existing channels (e.g., established regulatory bodies, laboratory collaborations) or through the creation of new mechanisms of responsible innovation [56]. Ethical reasoning can then be more closely linked to contexts of decision making, where concerns can shape real-world action. We see this not as a unique challenge for BCI ethics, but rather as a foundational difficulty faced by anyone working on emerging technologies, which can be both real objects in the world and to some extent a product of our imagination of possible futures.

Finally, and not disconnected from the two previous points, our results suggest a need for more empirical work. Our sample included a larger scale survey on the opinions of researchers regarding the ethics of BCI use and development [10]; focus groups of potential or current BCI users [9, 35, 38, 57, 58]; and studies of the general public’s opinions on BCI ethics, with rather small sample sizes (7 to 10 participants) [7]. Vlek et al., similarly, studied judgments of agency in the use of BCI [44]. These empirical studies, nonetheless, were the minority. Most authors in our sample employed non-empirical approaches, including conceptual analysis, analogical reasoning, and more or less informed speculation. This empirical gap has been noted before: BCI researchers responding to the Asilomar survey agreed that ethical issues must be empirically investigated [10], and the public participants in Jebari and Hansson’s (2013) convergence seminars unanimously encouraged future public participation on the social and ethical issues of BCI: “a need clearly exists for public participation in discussions regarding how and to what ends [BCI] should be used and on the risks and the ethical issues associated with it.” [7] Obtaining clarity on the challenges of BCI technology, in addition to identifying potential solutions, will likely require further empirical investigation into public hopes or worries, and into the emerging domains of BCI application.

## Conclusion

We undertook a scoping review of the ethical issues discussed in interdisciplinary bioethics literature regarding BCIs. Major issues discussed were identified (i.e., safety, humanity and personhood, stigma, autonomy, research ethics, privacy and security, responsibility, and justice), and qualitative summaries of these issues were extracted from the literature. These descriptive results provide a preliminary list of challenges to be addressed in the development of new neural technologies. And although we focused on providing a descriptive account of this literature, our findings also suggest ways to improve future research in ethics. Specifically, our sample of the BCI ethics literature, though substantial in content, tends towards generality, provides minimal practical guidance, and is lacking in empirical evidence. We interpret these features as typical for an emerging area of bioethics research. Looking ahead, we observe that researchers investigating the ethics of BCI have the opportunity, yet unfulfilled, to confront the challenges of BCI in greater detail and in parallel with empirically-oriented investigations.

## Additional file

Additional file 1: List of Coded Sources and Themes, By Source – Thematic table of sources (DOCX 38 kb)

## Abbreviations

BCI: Brain-computer interface

## Footnotes

1 A detailed depiction of sample characteristics, organized by article and ethical issue, is available in the supplementary data [additional file 1].

2 Note to readers: reference 37 was not part of the scoping review and is provided for explanatory purposes only.

3 Note to readers: reference 43 was not part of the scoping review and is provided for explanatory purposes only.

4 Interestingly, in articles that were beyond the scope of our inclusion criteria, we noted that BCI researchers tend to think about autonomy differently than ethicists do. Those researching and developing BCI technology tend to cite increased autonomy and quality of life in severely disabled individuals as the main goal of their research; for example, see [19, 20]. When they refer to autonomy, they are referring to the improved ability that these patients have for movement and communication with the use of BCI. In contrast, ethicists typically view autonomy through a philosophical lens as the capacity for intentional action without controlling external influences, see for example [32].
