# Supplementary material for: Ethical aspects of brain computer interfaces: a scoping review
Source: BMC Med Ethics. 2017 Nov 9;18:60. doi: 10.1186/s12910-017-0220-y (PMC5680604; doi:10.1186/s12910-017-0220-y)
Supplement: Additional file 1: — List of Coded Sources and Themes, By Source – Thematic table of sources (DOCX 38 kb) [file 12910_2017_220_MOESM1_ESM.docx]

| Year | Author(s) | Title | Type | | Ethical Issue Mentioned in Reference | | | | | | | | | | |
| --- | --- | --- | --- | --- | --- | --- | --- | --- | --- | --- | --- | --- | --- | --- | --- |
|  |  |  | Empirical | Non-empirical | Safety | Justice | Privacy & security | Risk-benefit | Personhood: identity | Personhood: human nature | Research ethics: informed consent | Responsibility | Autonomy | Research ethics: other | Stigma |
| 2005 | Farah MJ | Neuroethics: the practical and the philosophical |  | X | X | X | X |  | X | X |  | X |  |  |  |
| 2006 | Kubler A, Mushahwar VK, Hochberg LR, Donoghue JP | BCI meeting 2005--workshop on clinical issues and applications |  | X | X |  |  | X |  |  |  |  |  | X |  |
| 2006 | Wolpaw JR, Loeb GE, Allison BZ, Donchin E, do Nascimento OF, Heetderks WJ, Nijboer F, Shain WG, Turner JN | BCI meeting 2005--workshop on signals and recording methods |  | X | X |  | X | X |  | X |  |  |  |  |  |
| 2008 | Alpert S | Brain-computer interface devices: risks and Canadian regulations |  | X | X |  |  |  |  |  |  |  |  |  |  |
| 2008 | Fenton A, Alpert S | Extending our view on using BCIs for locked-in syndrome |  | X | X | X |  | X |  |  | X |  | X |  |  |
| 2009 | Haselager P, Vlek R, Hill J, Nijboer F | A note on ethical aspects of BCI |  | X | X | X | X | X |  |  | X |  |  | X |  |
| 2009 | Tamburrini G | Brain to computer communication: ethical perspectives on interaction models |  | X | X | X | X | X | X | X | X | X | X |  |  |
| 2009 | Clausen J | Man, machine and in between |  | X | X | X |  |  | X |  |  |  |  |  | X |
| 2010 | Kotchetkov IS, Hwang BY, Appelboom G, Kellner CP, Connolly ES, Jr. | Brain-computer interfaces: military, neurosurgical, and ethical perspectives |  | X | X | X |  | X |  |  | X |  |  |  |  |
| 2010 | Demetriades AK, Demetriades CK, Watts C, Ashkan K | Brain-machine interface: the challenge of neuroethics |  | X |  |  |  | X |  | X |  | X |  |  |  |
| 2011 | Hildt E | Brain-computer interaction and medical access to the brain: individual, social and ethical implications |  | X | X |  | X | X | X | X | X | X | X |  |  |
| 2011 | Clausen S | Conceptual and ethical issues with brain-hardware interfaces |  | X | X |  | X | X | X | X | X | X |  | X |  |
| 2011 | Tamburrini G, Mattia D | Disorders of consciousness and communication. Ethical motivations and communication-enabling attributes of consciousness |  | X | X |  |  | X |  |  |  |  | X | X |  |
| 2012 | Blain-Moraes S, Schaff R, Gruis KL, J. E. Huggins and P. A. Wren | Barriers to and mediators of brain-computer interface user acceptance: focus group findings | **X** |  | X |  |  |  | X |  |  |  | X |  |  |
| 2012 | Vlek RJ, Steines D, Szibbo D, Kubler A, Schneider MJ, Haselager P, Nijboer F | Ethical issues in brain-computer interface research, development, and dissemination |  | X | X | X | X |  |  |  | X |  |  |  |  |
| 2013 | McGie S, Nagai M, Artinian-Shaheen T | Clinical ethical concerns in the implantation of brain-machine interfaces: part II: Specific Clinical and Technical Issues Affecting Ethical Soundness |  | X | X |  |  |  |  |  | X |  |  |  |  |
| 2013 | McGie S, Nagai M, Artinian-Shaheen T | Clinical ethical concerns in the implantation of brain-machine interfaces: part I: overview, target populations, and alternatives |  | X | X |  |  | X | X | X |  |  |  |  |  |
| 2013 | Wolbring G, Diep L, Yumakulov S, Ball N, Leopatra V, Yergens D | Emerging therapeutic enhancement enabling health technologies and their discourses: what is discussed within the health domain? | **X** |  |  | X |  |  |  |  | X |  |  |  | X |
| 2013 | Jebari K, Hansson SO | European public deliberation on brain machine interface technology: five convergence seminars | **X** |  | X | X | X | X | X | X |  |  | X |  | X |
| 2013 | Moreno JD | Mind wars. Brain science and the military |  | X | X |  | X |  |  |  |  |  |  |  |  |
| 2013 | Evers K, Sigman M | Possibilities and limits of mind-reading: a neurophilosophical perspective |  | X |  |  | X |  |  |  |  |  |  |  |  |
| 2013 | Nijboer F, Clausen J, Allison BZ, Haselager P | The Asilomar survey: stakeholders' opinions on ethical issues related to brain-computer interfacing | **X** |  | X | X | X | X | X | X | X | X |  | X |  |
| 2013 | Purcell-Davis A | The representations of novel neurotechnologies in social media: five case studies | **X** |  | X | X |  |  |  |  |  |  |  | X |  |
| 2014 | Vlek R, van Acken J, Beursken E, Roijendijk L, Haselager P | BCI and a user's judgment of agency | **X** |  |  |  |  |  |  |  |  | X |  |  |  |
| 2014 | Carmichael C, Carmichael P | BNCI systems as a potential assistive technology: ethical issues and participatory research in the BrainAble project |  | X | X | X | X |  |  |  | X |  |  | X | X |
| 2014 | O'Brolchain F, Gordijn B | Brain-computer interfaces and user responsibility |  | X |  |  | X | X | X |  |  | X | X |  |  |
| 2014 | Birbaumer N, Gallegos-Ayala G, Wildgruber M, Silvoni S, Soekadar SR | Direct brain control and communication in paralysis |  | X |  |  |  | X |  |  | X |  |  |  |  |
| 2014 | Glannon W | Neuromodulation, agency and autonomy |  | X |  |  | X | X |  |  |  | X | X |  |  |
| 2014 | Grubler G, Hildt E | On human-computer interaction in brain-computer interfaces | **X** |  |  |  |  |  | X | X |  |  |  |  |  |
| 2014 | Huggins JE, Wolpaw JR | Papers from the fifth international brain-computer interface meeting. Preface |  | X |  | X |  |  |  | X |  |  | X |  |  |
| 2014 | Tamburrini G | Philosophical reflections on brain-computer interface |  | X | X |  |  | X |  | X | X | X | X |  | X |
| 2014 | Rose N | The human brain project: social and ethical challenges |  | X |  | X | X |  |  |  |  |  |  | X |  |
| 2014 | Varios Authors | The users' perspective | **X** |  |  |  |  |  |  |  |  |  |  |  |  |
| 2015 | Bonaci T, Calo R, Chizeck HJ | App stores for the brain: privacy and security in brain-computer interfaces |  | X |  |  | X |  |  |  |  | X |  | X |  |
| 2015 | Aas S, Wasserman D | BCIs and disability: extending embodiment, reducing stigma |  | X |  |  |  |  | X |  |  |  |  |  | X |
| 2015 | Klein E, Brown T, Sample M, Truitt AR, Goering S | Engineering the brain: ethical issues and the introduction of neural devices |  | X |  | X | X |  | X |  |  | X |  |  | X |
| 2015 | Farisco M, Laureys S, Evers K | Externalization of consciousness. Scientific possibilities and clinical implications |  | X |  |  |  |  |  |  |  |  | X | X |  |
| 2015 | Klein E | Informed consent in implantable BCI research: identifying risks and exploring meaning |  | X | X | X | X | X | X |  | X | X |  | X | X |
| 2015 | Schicktanz S, Amelung T, Rieger JW | Qualitative assessment of patients' attitudes and expectations toward BCIs and implications for future technology development | **X** |  | X | X | X | X |  | X |  |  | X |  | X |
| 2015 | Nijboer F | Technology transfer of brain-computer interfaces as assistive technology: barriers and opportunities | **X** |  |  | X |  |  |  |  |  |  |  | X |  |
| 2015 | Zehr EP | The potential transformation of our species by neural enhancement |  | X |  | X |  |  |  | X |  |  |  |  | X |
| 2016 | Wolbring G, Diep L | Cognitive/neuroenhancement through an ability studies lens. | **X** |  |  | X |  |  | X |  |  |  |  |  | X |
| Totals | | 42 | **11** |  | **24** | **20** | **19** | **19** | **15** | **14** | **14** | **13** | **12** | **12** | **11** |
